# Advances in and Perspectives on Transgenic Technology and CRISPR-Cas9 Gene Editing in Broccoli

**DOI:** 10.3390/genes15060668

**Published:** 2024-05-23

**Authors:** Li Zhang, Sufang Meng, Yumei Liu, Fengqing Han, Tiemin Xu, Zhiwei Zhao, Zhansheng Li

**Affiliations:** 1 State Key Laboratory of Vegetable Biobreeding, Institute of Vegetables and Flowers, Chinese Academy of Agricultural Sciences, Beijing 100081, China; zl1999202106@163.com (L.Z.); hnnydxmsf@163.com (S.M.); liuyumei@caas.cn (Y.L.); hanfengqing@caas.cn (F.H.); xutiemin@caas.cn (T.X.); 2 College of Horticulture, Shanxi Agricultural University, Taigu 030801, China; 3 Shouguang R&D Center of Vegetables, CAAS, Shouguang 262700, China; zgys9814@126.com

**Keywords:** broccoli, advances, gene editing, CRISPR-Cas9, *Agrobacterium*-mediated transformation

## Abstract

Broccoli, a popular international *Brassica oleracea* crop, is an important export vegetable in China. Broccoli is not only rich in protein, vitamins, and minerals but also has anticancer and antiviral activities. Recently, an *Agrobacterium*-mediated transformation system has been established and optimized in broccoli, and transgenic transformation and CRISPR-Cas9 gene editing techniques have been applied to improve broccoli quality, postharvest shelf life, glucoraphanin accumulation, and disease and stress resistance, among other factors. The construction and application of genetic transformation technology systems have led to rapid development in broccoli worldwide, which is also good for functional gene identification of some potential traits in broccoli. This review comprehensively summarizes the progress in transgenic technology and CRISPR-Cas9 gene editing for broccoli over the past four decades. Moreover, it explores the potential for future integration of digital and smart technologies into genetic transformation processes, thus demonstrating the promise of even more sophisticated and targeted crop improvements. As the field continues to evolve, these innovations are expected to play a pivotal role in the sustainable production of broccoli and the enhancement of its nutritional and health benefits.

## 1. Introduction

Broccoli (*Brassica oleracea* L. var. *italica*) is a cruciferous vegetable from the Brassica family. Also referred to as green cauliflower or broccoli, it is cherished for its rich nutritional profile, which includes a spectrum of vitamins, proteins, minerals, antioxidants, and phenolic compounds [1,2,3,4,5]. Also known as the “Crown of Vegetables”, broccoli is a global favorite food due to its numerous health benefits. Broccoli contains lycopene sulfide, which is a compound with anticancer properties that may also effectively prevent myopia, cataracts, and cardiovascular diseases [6]. Additionally, it has been shown to improve hypertension, ameliorate certain complications related to type 2 diabetes, reduce cholesterol levels, and foster the growth of beneficial gut microbiota [2,7]. Broccoli’s contribution to weight and fat loss, enhanced metabolism, and strengthened immunity further solidifies its status as a healthful food choice [8,9]. Broccoli is also noted for containing the compound sulforaphane, which inhibits the growth of cancer cells and induces apoptosis in some cancers [10,11,12,13,14,15].Recent studies have also highlighted the unique antiplatelet selectivity of sulforaphane, which can reduce thrombus formation and enhance the thrombolytic activity of recombinant tissue plasminogen activator (rtPA), thus exhibiting promise for improved preventive and therapeutic strategies [16]. Moreover, phenolic compounds extracted from broccoli have shown efficacy in preventing and reducing fatty liver formation [17]. Antioxidant extracts with anti-inflammatory properties, such as radicicchioidin, indole-3-methanol, isothiocyanates, flavonoids, and quercetin, can mitigate inflammation by inhibiting enzymatic activity [18,19]. Certain compounds in broccoli, including thioglucosides and isothiocyanates, have demonstrated antimicrobial activity, with sulforaphane potentially acting against *Helicobacter pylori* [20,21,22]. Additionally, indole-3-methanol and phenolic compounds exhibit inhibitory effects on specific bacterial strains, such as *Escherichia coli* and *Staphylococcus aureus* [18]. In summary, the diverse health benefits of broccoli, which are supported by scientific research, make it a powerful supplement to any diet. Its potential to combat a range of health issues, ranging from cancer to cardiovascular diseases, underscores its importance as a nutritional powerhouse food.

**Figure 1:**
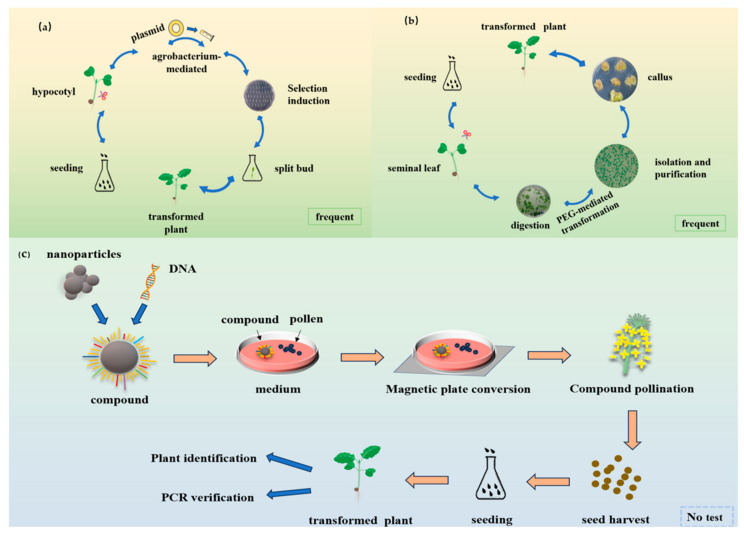
(**a**) The profile of *Agrobacterium-mediated* genetic transformation; (**b**) the profile of PEG-mediated genetic transformation of protoplasts; (**c**) the process of pollen magnetotransfection in plants.

Genetic transformation is a phenomenon in which a cell of a specific genotype absorbs DNA from a cell of a different genotype that is present in its environment, resulting in a change to its own genotype and gene expression. This process, known as genetic transformation, involves the uptake of homologous or heterologous DNA molecules by a receptor cell, which can occur naturally or be induced artificially. These DNA molecules can originate from natural sources or can be artificially created, and their incorporation into the receptor cell leads to the horizontal transfer and expression of genes. Genetic transformation can be categorized into natural and artificial transformation, with the former type being a physiological feature of a cell at a particular growth stage and the latter type involving artificial methods to induce DNA uptake or directly introduce DNA into the cell. *Agrobacterium*-mediated genetic transformation is a technique that involves isolating target gene fragments, constructing a vector to hold these fragments, and then using *Agrobacterium* in a sensitive state to transfer the vector into plant cells. Genetic transformation includes recombinant DNA technology, cell tissue culture technology, germplasm system transformation technology, and so on (Figure 1). The target gene is integrated into the plant genome, and the resulting transgenic plants are verified for the presence and expression of the gene [23,24,25,26]. Genome editing technologies allow for the introduction of desired traits into crop varieties through targeted mutagenesis and precision breeding. These technologies enable the simultaneous editing of multiple genetic loci, thus facilitating the rapid accumulation of important traits [27,28,29]. To date, genome editing has been applied to a broad array of crops, including cereal grains such as maize [30,31], wheat [32], sorghum [33,34], barley [35], sugarcane [36,37], rye [38], millet [39], and various vegetables within the cruciferous family, such as Chinese cabbage [40,41], Chinese Kale [42,43], broccoli [26,44], and carrot [45]. Additionally, genome editing has been extended to other crop species, such as eggplant [46], chilli [47], tomatoes [48,49], and carrots in the umbelliferae family [50,51]. The application of these technologies in crop improvement continues to expand, offering new possibilities for enhancing agricultural productivity, sustainability, and the nutritional value of food crops.

The expression of genes can vary based on the genetic transformation technology that is used, the genotypes involved, the species of the organism, and the specific body part where the gene is being expressed. By manipulating genes through knockout (deleting a gene), overexpression (increasing gene expression), or gene silencing (reducing gene expression), researchers can elicit a range of phenotypic responses, resistances, and functional changes in the organism. As genetic transformation technologies continue to evolve and improve, we can anticipate significant advancements in plant breeding. These breakthroughs will not only enhance agricultural productivity but also drive the development of new crop varieties with improved traits, such as increased yield, disease resistance, drought tolerance, and enhanced nutritional content. The refinement of these techniques will play a crucial role in addressing global food security challenges and fostering sustainable agricultural practices.

Somatic cell fusion has made significant strides in both food and cash crops, thus enabling the integration and exchange of genes between protoplasts of different genera. This technique allows for the breaking of barriers to intergeneric hybridization and the creation of new varieties with varying ploidy levels, improved resistance, and enhanced agronomic traits and quality [52]. There are two primary methods of somatic cell fusion: physical (electrofusion) and chemical (PEG fusion) fusion [53]. The process of somatic cell fusion encompasses several steps, including the extraction and purification of protoplasts, the fusion of protoplasts, the culture and regeneration of fused protoplasts, and the identification of hybrids [54,55,56].

Reports have described the successful generation of hybrid tobacco plants with different genotypes through protoplast fusion [57]. Similarly, new hybrids have been obtained by fusing celery protoplasts with those of carrot, coriander, and white celery [58]. Cytoplasmic male sterile plants are produced by fusing the cytoplasm of *Brassica napus* with the nucleus of oilseed rape [59]. Protoplasts from green cauliflower pollen and leaf pulp were fused by using PEG to obtain hybridized callus tissue [60]. The transfer of Polima-type cytoplasmic male sterility (CMS) from kale-type oilseed rape to *Brassica napus* has been achieved by using protoplast fusion to create nuclear hybrids [61]. Complete novel genomic plants have been created by fusing eggplant and tomato protoplasts [62]. After protoplast fusion in oilseed rape, the resulting plants displayed new cytoplasmic morphological markers [63]. Interspecific somatic hybrids have been obtained by fusing protoplasts of rice and barley [64], and asymmetric crosses between wheat and *Arabidosis thaliana* have yielded regenerated callus tissue and green plants resembling wheat [65]. Seven hybrids of kale and white rapeseed were obtained through the electrofusion of protoplasts [66]. Additionally, there has been notable progress in somatic cell hybridization within the Brassica crops of the Cruciferae family and citrus crops of the Brassicaceae family [67,68,69,70,71,72]. By pairing different varieties and regenerating plants, researchers have been able to introduce new traits and improve crop productivity. As somatic cell fusion techniques continue to advance, they exhibit promise for the development of innovative crop varieties that can meet the demands of a growing population and adapt to the challenges of climate change [73].

Transient expression in protoplasts has emerged as an efficient and straightforward technique due to advancements in technology. It offers several advantages over other transformation methods, including lower costs, higher efficiencies, and greater flexibility. Protoplast transformation does not require specialized equipment or species-specific adaptations, and it allows for the rapid acquisition of validation results [74,75,76]. Protoplasts typically maintain the cellular identity and differentiation state of their parent cells, which is a crucial factor in the design of genetic function screens [77]. The process involves introducing exogenous genes into protoplasts of various species via polyethylene glycol (PEG) induction, and the transformation efficiency is assessed via laser confocal microscopy. Once protoplasts are successfully transformed, they can be cultured to regenerate transgenic plants, and the system for protoplast transformation and regeneration continues to evolve [55,74]. The pioneering work in this field dates back to 1960, when Cocking first reported of the isolation of protoplasts; moreover, in 1969, Aoki and Takebe achieved the successful transformation of TMV in tobacco protoplasts [78].

Over the past few decades, many crops have been successfully transformed and regenerated by using protoplasts. Transformation can be achieved through various methods, including electroporation, PEG-induced transformation, and *Agrobacterium*-mediated delivery. These include model plants such as *Arabidopsis thaliana* [74,79,80,81], and a range of food crops such as tomatoes [82,83], cabbage [84,85], broccoli [86], carrots [87,88], maize [89,90,91], soybeans [92,93,94], and rice [95], as well as cash crops such as sugarcane [96], bananas [97], strawberries [98,99], and apples [100]. Although genetic transformation technologies, somatic cell fusion, and protoplast transient expression have advanced in research related to *Brassica napus*, there is a relative lack of comprehensive reviews focusing on the specifics of genetic transformation and gene editing in this important crop. As the field continues to progress, it will become increasingly important to synthesize and analyze the findings from these various techniques to enhance our understanding and application of genetic engineering in *Brassica napus* and other crop species.

## 2. Construction and Optimization of the Genetic Transformation System for Broccoli

Genetic transformation methods, including *Agrobacterium tumefaciens*, gene gun, floral dip, polyethylene glycol (PEG), protoplast transformation, and free microspore culture, have been used for cruciferous crops for the past 40 years [45,101,102]. The main methods of genetic transformation research in broccoli have focused on *Agrobacterium tumefaciens*-mediated transformation and protoplast transformation methods [103]. *Agrobacterium tumefaciens*-mediated genetic transformation is widely used for model plants and field crops [25,104,105]. The efficient instantaneous transformation of protoplasts has only improved and been established in broccoli in recent years. Callus-free protoplasts have been used to create regenerated plants in cereal crops such as corn and rice [106]; however, this method has not been used for *Brassica oleracea* crops such as broccoli, cabbage, cauliflower, and Chinese kale [107,108].

It has been reported that the transformation efficiency of the *Agrobacterium tumefaciens*-mediated genetic transformation system for broccoli is generally approximately 2–26%, which is influenced by the explant type, receptor genotype, hormone concentration, culture time, and other factors [109,110]. In general, the broccoli genotype is the main factor affecting the efficiency of genetic transformation [44,109]. It has been established that when the concentration of hygromycin B is 5 mg/L for the generation of young sprouts, significant differences in the differentiation rate are usually detected among broccoli genotypes. Moreover, the vector type has also been shown to affect the differentiation rate of broccoli [111]. The selection of a better receptor material with a greater differentiation and regeneration rate is very important for the verification and identification of gene functions, which has been proven in broccoli, such as in the inbred line B42 [110].

In recent decades, the systematic genetic transformation mediated by *Agrobacterium tumefaciens* has been constructed and developed based on broccoli hypocotyls [112] (Table 1). Most research has shown that the selective antibiotic hygromycin B is most widely used in broccoli and has the highest and most stable selection efficiency. According to comparisons with other *Brassica* crops, such as *Brassica napus* and *Brassica rapa*, the optimal selection concentration is 5 mg/L hygromycin B in broccoli [109]. In addition, 8 mg/L hygromycin B is better for cabbage, whereas 50 mg/L hygromycin B is generally used for rape [113].

## 3. Research and Application of Genetic Transformation Technology in Broccoli

### 3.1. *Agrobacterium Tumefaciens*-Mediated Genetic Transformation System

#### 3.1.1. Agronomic Traits

Several studies of agronomic traits in broccoli have been reported. Ogura cytoplasmic male sterility (Ogura CMS) is widely used in the breeding of broccoli and other *Brassica oleracea* crops [127,128]. The Ogura CMS restorer gene *Rfo* from *Brassica napus* was successfully introduced into broccoli, and the Rfo transgenic plants tended toward broccoli and showed significant genetic segregation [129]. The cotyledons were subsequently transformed with *Agrobacterium* to obtain 20 transgenic plants; moreover, the transformed plants exhibited normal male sterility, and fertility was restored by using exogenous jasmonic acid treatment [114]. MicroRNA171 (miR171) functions in plant growth and development, hormone signaling, and stress responses; additionally, it plays important roles in plants through interactions with microbes and other small RNAs, and its overexpressing transgenic broccoli (*Bol-miR171b*) plants have dark green leaves because of increased chlorophyll levels. Additionally, all of the flowers are nearly sterile [115].

#### 3.1.2. Quality Traits

Broccoli is rich in glucoraphanin, the second product of which is sulforaphane, which is beneficial to human health, has anticancer effects, and is a hot topic and research field in medicine, biochemistry, and agriculture [130,131,132,133,134,135,136]. According to the overexpression of the P450 gene *CYP79F1* in broccoli, *BoCYP79F1* can significantly upregulate the production of sulforaphane and glucoraphanin at the bolting stage, which is consistent with previous reports [137]. An analysis of 80 broccoli genotypes and eight developmental organs demonstrated that small flowers contain 12 glucosinolates, ranging from 0.467 to 57.156 µmol/g DW. The glucosinolate content in the roots accounted for 43% of the total content, and the glucosinolate content in different organs accounted for 29% of the total content. By examining the changes in glucosinolate profiles in 80 genotypes and eight developmental organs, the correlation between glucosinolates and both genotypes and organs was determined. Statistical analysis of glucosinolates indicated that glucosinolate components in roots differ from those in other nutrient organs [138]. To obtain plants with high sulforaphane content, *MAM1*, *FMOGS-OX2*, and black mustard enzymes, which are required for SF biosynthesis, were introduced into *Brassica napus* via *Agrobacterium*-mediated transformation. The results showed that the SF content increased 1.86–5.5-fold in plants transformed with the three transgenes compared with that in the wild type (WT) [116]. Studies have suggested that *BroMYB28* may play a role in glucoraphanin synthesis [139]. Moreover, according to *Agrobacterium*-mediated transient overexpression of MYB28, *BoMYB28* has been shown to play a role in aliphatic glucosinolate synthesis in broccoli, which is similar to that in *Arabidopsis* [117].

#### 3.1.3. Biotic and Abiotic Stress

Currently, extreme weather and climate conditions, including high temperatures, cold damage, droughts, floods, and haze, which usually threaten the growth and development of broccoli, frequently occur [130]. The cuticle wax on the surface of plants helps plants to resist many environmental stresses, such as drought, ultraviolet radiation, high radiation, and bacterial and fungal pathogens; moreover, some loci and linkage markers for this trait have been identified [110]. An RNAi transgenic blue and white kale line was generated by targeting the cellulose synthase gene *BoiCesA*; consequently, the cellulose content was reduced, salt tolerance was enhanced, and the expression of related genes was significantly altered, as evidenced by dwarfing and thinning leaves [118]. The yield of cultivated plants is influenced by natural environmental factors, and soil salinization has become an increasingly serious global problem (ionic stress) [140]. The CCCH-type (C3H-type) zinc finger (Znf) protein, which contains a typical motif with three cysteine residues and one histidine residue, plays an important role in plant growth stress. *BoC3H* has been proven to potentially improve salt stress tolerance by regulating free proline and MDA in broccoli plants overexpressing this gene [119]. Moreover, transgenic plants showed enhanced tolerance to salt stress after overexpression of BoC3H4 in broccoli, indicating that BoC3H4 is a positive regulator of salt stress tolerance in plants [120]. The gene encoding the *ERF* transcription factor *BoERF1* was isolated from *Brassica napus*, and *BoERF1*-overexpressing transgenic plants were generated via *Agrobacterium*-mediated transformation. The transgenic plants showed greater seed germination and less chlorophyll loss under salt stress than the wild-type (WT) *Brassica napus* plants. These results indicate that *BoERF1* plays a positive role in salt stress and stem rot resistance in nuclear mycelia, suggesting its potential application value in the molecular breeding of *Brassica napus* [121].

Common diseases in broccoli include black rot, downy mildew, root swelling, viral diseases, wilt, black spot, and soft rot, among other diseases. Currently, research on broccoli has mainly focused on downy mildew, black rot, and root swelling [130]. Broccoli downy mildew is a common pathological disease caused by downy mildew fungi in cruciferous plants [141]. The *BoDFN* gene has been shown to increase the resistance of broccoli to downy mildew via upregulation of the expression level of this gene [122]. Another gene known as *BoWRKY6* also plays a positive role in the downy mildew resistance of broccoli, and two positive broccoli strains have strong resistance to downy mildew [123]. In total, six transgenic broccoli lines were obtained by introducing *RsrSOD* via *Agrobacterium*-mediated transformation; these plants exhibited the greatest resistance to downy mildew, which is beneficial for broccoli breeding [124]. Seven transformed plants overexpressing the *BoAPX* gene were obtained via *Agrobacterium* transformation. Four genetic strains were extremely resistant to downy mildew, including *apx07*, *apx15*, *apx32*, and *apx33*, which are highly tolerant to heat stress and play important roles in cellular defense against ROS-mediated oxidative damage [125].

The black rot pathogen can easily overwinter in soil or seeds. The pathogen prefers high temperature and high humidity and is susceptible to disease in hot and rainy weather or under continuous cropping conditions [142]. At present, domestic and foreign research on the mechanism of black rot resistance is limited, and there is a lack of broccoli varieties with high resistance to black rot. Due to the fact that there are many sources of resistance in the A and B genomes of *Brassica juncea*, resistance genes can be introduced into broccoli through distant hybridization [110].

In recent years, clubroot disease has become more common among broccoli, cauliflower, and other cruciferous vegetable crops in some provinces in China, such as Yunnan, Hubei, and Zhejiang [143,144]. Dominant clubroot resistance (CR) genes, such as *CRa*, *CRb*, *CRk*, and *Crr1-3*, have been found in Brassica crop species, including Chinese cabbage, *Brassica napus*, *Brassica rapa*, and radish, which provides insights into the utilization of broccoli. Cabbage has been successfully introduced into the *CRb* gene, resulting in increased resistance to *Plasmodiophora brassicae* Race 4 by backcrossing with *Brassica napus* [145,146]. However, there are still no reports on transgenic CR broccoli.

The pests infecting broccoli include the diamondback moth, cabbage worm (*Pieris rapae*), and cotton bollworm. The *Bacillus thuringiensis* (Bt) gene is the most extensively studied and used gene in broccoli and other *Brassica oleracea* crops. The Bt genes *Cry1C*, *Cry1Ac*, *cryIA(b)*, *Cry2Ab12*, *Cry1la8*, and *Cry1Ba3* have been successfully transferred into broccoli and *Brassica napus* to improve toxicity to the diamondback moth [147,148,149]. *Cry2Ab12* is not lethal to the diamondback moth, but it can decrease the body weight of the diamondback moth by 33%. Insect biology experiments have demonstrated that the transgenic “Solan Green Head”, which is a *cryIAa*-containing strain of *Agrobacterium tumefaciens* that is resistant to codling moth larvae, is effective against codling moth infestation [150].

With the large-scale application of herbicides, there is an urgent need to cultivate herbicide-resistant broccoli to meet future demand. The results of the GUS staining of pBoGIR1-expressing broccoli transgenic plants have indicated that *pBoGIR1* can be used as an effective alternative to antibiotic and herbicide resistance genes for the cultivation of transgenic crops. In broccoli, there have been fewer studies on the genetic transformation of herbicides, which can be explored in-depth to understand the research on breeding herbicide-resistant varieties (US Patent Application for Application of the broccoli wound-inducible promoter of GLUCOSE INHIBITION of the ROOT ELONGATION 1 gene in transgenic plants Patent Application [Application #20140013472 issued 9 January 2014]—Justia Patents Search).

#### 3.1.4. Others

Nanomaterials have been an international research hotspot, and genetic transformation studies in the field of nanomaterials have been successful in maize [151], and cotton [152,153]. Exogenous DNA-loaded magnetic nanoparticles can be delivered into pollen, and transgenic plants can be successfully obtained from transformed seeds [154]. Pollen magnetization may facilitate the production and stress tolerance of transformed germplasms, but this phenomenon has not been reported in broccoli or other *Brassica oleracea* crops. This method will further facilitate germplasm innovation and the design of future crops (Figure 1).

### 3.2. Application of the Protoplast-Mediated Instantaneous Transformation System

Protoplast fusion is the process in which the protoplasts of two heterologous parents fuse with each other under appropriate conditions, thus further integrating intracellular substances to form hybrid cells. Therefore, protoplasts are widely used for genetic transformation and somatic cell fusion [52,55]. According to polyethylene glycol (PEG)-mediated protoplast fusion, hypocotyl protoplasts of kale were successfully hybridized with chloroplast protoplasts of black mustard, and 15 somatic hybrids were obtained from the callus [155]. The hypocotyl and cotyledon protoplasts of mustard and broccoli were fused by using 40% (*w*/*v*) polyethylene glycol, and the regenerated plants had normal petals and stamens; however, only two plants produced pollen [156]. By fusing the pollen protoplasts and haploid leaf pulp protoplasts of broccoli, 40% polyethylene glycol 4000 resulted in a maximum fusion frequency of approximately 20%. The results demonstrated the presence of a hybrid callus [60]. Using a 20% PEG 4000-mediated efficient instantaneous transformation of protoplasts, the subcellular localization of the resistance gene *CRa* and the gluraphanin metabolism-related gene *FMOGS-OX5* (*Bol029100*, *Bol031350*) was identified in broccoli [86,109]. Therefore, the PEG-Ca^2+^-mediated transformation of plant protoplasts has great advantages and application value in gene function analysis and breeding design [86]. However, there are still some problems, such as difficulty in the protoplast separation of special tissues and organs, as well as the low frequency of callus regeneration [157].

### 3.3. Others

In the genetic transformation of broccoli, the main method that is currently used is an efficient instantaneous transformation system mediated by *Agrobacterium tumefaciens* and protoplasts. In future research, we can apply the gene gun and floral dip methods in broccoli. The floral dip method is simple to execute and has high transformation efficiency. After transformation, transgenic plants can be directly generated, thus avoiding the need for somatic or protoplast culture and decreasing the survival rate of plants after transplantation. The dipping method has been successfully applied in multiple species, including in both cruciferous plants such as radish and salt mustard but also in important crops such as wheat and cash crops, including legumes [158]. There is limited research on broccoli, given its low efficiency or inability to achieve efficient genetic transformation due to genomic differences.

## 4. Research and Application of Gene Editing Technology in Broccoli

Genome editing refers to the precise design and efficient transformation of organisms at the genomic scale. Genome editing techniques include the use of meganucleases, zinc finger nucleases (ZFNs), transcription activator-like effector nucleases (TALENs) [159,160], and clustered regular interval short palindrome repeat (CRISPR) systems. CRISPR-Cas9 is currently the most widely used and efficient plant genome editing method [161,162,163,164,165,166]. In recent years, this genome editing system has been successfully applied to various crops, including rice, corn, soybeans, and tomatoes, for gene function identification and crop improvement, thus resulting in several ideal traits, such as increased yield, disease resistance, and herbicide resistance. However, there are still few reports on this topic in broccoli [110].

It has been reported that the accumulation of glucoraphanin in broccoli leaves and flower buds is decreased by knocking out MYB28, which also results in a decrease in the amount of aliphatic glucosinolate in broccoli [167]. The cycad genotype 19B42 was found to have high regenerative capacity and is suitable for transformation [109]. A recent study reported that the dominant gene *BoGL5* can positively regulate wax synthesis in broccoli, and CRISPR-Cas9 validation demonstrated that the functional loss of this gene leads to the absence of wax, thus resulting in glossy mutants of broccoli [126]. The abovementioned research indicates that the CRISPR-Cas9 system can effectively induce gene-specific mutations in the broccoli genome, which is highly important for further verifying genetic functions and enabling precise trait improvements in broccoli.

## 5. Future Perspectives

As extreme weather events, diseases, and pests become more prevalent, global crop breeding has become increasingly recognized as being a critical trend for the future, thus necessitating a worldwide call to enhance crop resilience in the face of climate change. The development of new broccoli varieties capable of adapting to future climatic conditions is of utmost importance. To expedite the transformation and breeding of broccoli, it is essential to innovate genetic transformation methods that are less dependent on specific genotypes. Additionally, enhancing the efficiency of *Agrobacterium*- and PEG-mediated transient transformation in broccoli is crucial. Experiments with new bacterial strains that exhibit high infection efficiency and the use of specific nanomaterials for transformation are potential avenues to explore in broccoli genetic engineering [168,169]. Furthermore, significant advancements in ternary vector systems for *Agrobacterium*-mediated transformation have been made, which are vital for gene function analysis and the creation of novel germplasms [170,171,172]. These developments underscore the need for a global commitment to crop improvement as a means to counteract the challenges posed by climate change. In the future, it is anticipated that continued optimization and innovation of technologies will be targeted at crop enhancement, thus aiming to improve flavor, quality, and resistance and to provide superior breeding materials. These efforts will not only improve food security but also contribute to the sustainability of agricultural systems worldwide.

## Figures and Tables

**Table 1:** Current information of genetic transformation genes in broccoli.

Gene Transferred	Exosome Type		Expression Vector Types			Functional Information	References
	Seminal leaf	Hypocotyls	CRISPR- Cas9	Over- expressing	RNAi		
*BoiDAD1F*	√				√	recoverable male sterility	[114]
*Bol- miR171b*	√			√		nearly completely male sterile and increased the chlorophyll content	[115]
*BoMAM1*	√			√		led to an increase in SF content	[116]
*BoFMOGS-OX2*	√			√		led to an increase in SF content	[116]
*BoMyrosinase*	√			√		led to an increase in SF content	[116]
*BroMYB28*	√			√		increased glucoraphanin content	[117]
*BoiCesA*	√				√	enhanced salt tolerance; dwarf and smaller leaves	[118]
*BoC3H*	√			√		enhanced salt stress tolerance	[119]
*BoC3H4*	√			√		enhanced salt stress tolerance	[120]
*BoERF1*	√			√		enhanced salt stress tolerance; enhanced resistance to Sclerotinia stem rot	[121]
*BoDFN*	√			√		downy mildew resistance	[122]
*BoWRKY6*	√			√		downy mildew resistance	[123]
*RsrSOD*		√		√		downy mildew resistance	[124]
*BoAPX*	√			√		enhanced resistance to downy mildew enhanced tolerance to heat stress	[125]
*BoGL5*		√	√			mutants lacked cuticular waxes	[126]

## Data Availability

The data presented in this study are available upon request from the corresponding author.
